# Screening of the unrecognised bipolar disorders among outpatients with recurrent depressive disorder: a cross-sectional study in psychiatric hospital in Morocco

**DOI:** 10.11604/pamj.2017.27.247.8792

**Published:** 2017-08-03

**Authors:** Oneib Bouchra, Sabir Maria, Ouanass Abderazak

**Affiliations:** 1 Department of Psychiatry, Faculty of Medicine, University Mohammed I^er^, Oujda, Morocco; 2 Department of Psychiatry, Faculty of Medicine, University Mohammed V, Rabat, Morocco

**Keywords:** Recurrent depression, hypomania, bipolar disorder, unrecognised, misdiagnosis

## Abstract

The bipolar disorder is often misdiagnosed in particular among outpatients with recurrent depression. Indeed, this work confirmed that the unrecognised bipolar disorder is common among depressed outpatients, which were younger, unemployed, single or divorced with a low socio-economic level. These socio-demographics data gives us an idea about the disability experienced by the unknown bipolar patients. Also, we demonstrate that the under-diagnosis bipolar disorder was associated with the earliest onset age of a depressive episode and it was more prevalent in depressed patients with suicidal ideation and suicide attempts. These factors should be taken into account when we screen for the unknowm bipolar disorder, especially type II to improve the early diagnosis and the quality of life of these patients.

## Introduction

Prevalence of bipolar disorders on the whole life was estimated at 1% of the general population. However, it was demonstrated recently that this prevalence exceeded 5% [1, 2]. This variation in prevalence is explained by the long delay of diagnosis of bipolar disorder [3]. Indeed, these patients often had initially a major depressive episode and they were wrongly diagnosed as an unipolar depression in different proportions going from 30% to 50% [1]. Accordingly, it takes several years for patients to have the correct diagnosis [4]. Thus, delayed diagnosis could increase antidepressant-induced manic episodes, occur rapid cycling, resistance due to anti-depressant drugs and a highest risk of suicide [5, 6]. The unrecognised bipolar disorder, mostly observed among depressed outpatients in 10-45% [7]. Conforming to a study conducted between 1995 and 2000 using a large database, showed recognition of diagnosis of bipolar disorder in the proportion of 67% among depressed outpatients [8]. Currently, clinical discrimination of hypomanic symptoms among depressive patients is usually undetected, leading to under-diagnosis the bipolar disorder [9]. In fact, patients do not often report Hypomania and they perceive symptoms as a well-being and a productive state [10]. A Russian study revealed a high rate of patients meeting diagnostic criteria for bipolar disorder II among patient with a current diagnosis of recurrent depressive disorder (40.8%) [11]. The objective of our study was to estimate the prevalence of the unrecognised bipolar disorder among outpatients with recurrent depressive disorder, determinate the clinical features of depressive episodes of these patients and specify if there were any associated factors.

## Methods

We carried out a cross sectional study at a psychiatric hospital in Rabat, which receiving the majority of patients in the northwest region of Morocco, during six months (from February to July 2015). We included all outpatients beyond 18 years, filling the criteria of DSM IV of recurrent depression at the time of evaluation and French speaking. Of every patient, we obtained an oral consent after explaining the aim of the study. We excluded patient with known bipolar disorder, schizoaffective disorder, other mental disorder resulting from a general medical condition and any significant medical condition. As well as the subjects, which did not give their consent. Of the 250 outpatients recruited, only 101 patients met the inclusion criteria and accepted to participate in the study. Methods and instruments: we specified at first the sociodemographic characteristics of the participants in particular the age, the sex, the profession, the marital status and the socioeconomic level. In the second place, we determined some characteristics of the depressive episodes of these patients, especially the age of onset of the depressive disorder, the number of the episodes. Some clinical features were clarified by the validated Moroccan version of the Mini- International Neuropsychiatric Interview semi-structured (MINI) to evaluate the melancholic symptoms, the suicidal ideations and suicide attempts [12]. The diagnosis of recurrent depression was made by a psychiatrist using the MINI. After we completed the clinical assessment, we used the hypomania checklist of Angest translated and validated in French version to identify hypomania symptoms, patients were asked to complete this self-rating scale which containing 20 items. The hypomania checklist of Angest had a sensitivity of 82% with a specificity of 57% compared to current DSM-IV criteria for bipolar disorder [9, 13]. A French multi-center study (EPIDEP) showed that a score above 10 towards a diagnosis of hypomaniac episode, allowed the distinction between the unipolar and bipolar disorder [14]. Statistical analysis: the statistical analysis was realized by the software SPSS 20.0 Windows. A descriptive analysis of the various parameters was made. The quantitative variables were described as a means ± standard deviation and the qualitative variables were expressed in percentages. To search the associated factors to the hypomania, we used the test t Student and test chi 2. Then a logistic regression was used to take into account possible confounder factors. A p< 0.05 was considered as significant.

## Results

**The characteristics of sample**: A total of 101 outpatients who met the inclusion and exclusion criteria, the majority of participants were women and 15% of outpatients had a history of psychiatric hospitalisation (Table 1).

**Table 1:** Sociodemographic characteristics

Characteristics	N (%)
Age* (years)	42.5 ± 11
**Gender**	
Man	28(27.8)
Women	73(72.2)
**Marital status**	
Couple	67(67.2)
Live alone	34(33.8)
**Profession**	
With	56(56.4)
Without	47(44.6)
**Socioeconomic level**	
Low	42(42)
Medium	52(52)
High	6(6)

* Means ± deviation standard

**Prevalence and clinical characteristics of undiagnosed bipolar disorder**: According to the hypomania checklist Angest scale, 22% of outpatients met the criteria for bipolar disorder. Previously undiagnosed bipolar disorder patients were relatively younger with a mean age of 37.3 ans ± 7 years. Approximately 82% of these patients were women, 60% were unemployed, 31% were single or divorced and 66% had a low socioeconomic level. The outpatients identified with bipolar disorder did more depressive episodes beyond than three episodes (35%), with more melancholic features. These patients had earlier onset of recurrent depression with a mean age of 24.5, on the other hand unipolar patients had a mean age of 36.29. Also, undiagnosed bipolar disorder patients thought more to suicide (90%) and more than half of them attempted to suicide.

**Analytical statistics**: Univariate analysis revealed that only early age of onset of depressive episodes was associated with the undiagnosed bipolar disorder. So multiple logistic regression analysis was not conducted.

## Discussion

The bipolar disorder, especially II is often misdiagnosed. Only 20% of bipolar patients received the correct diagnosis, whereas 31.2% had received a diagnosis of unipolar depression [2]. The EPIDEP study revealed that just the half of patients was recognized by the clinicians as having bipolar disorder [15]. In our knowledge, this is the first investigation in Morocco concerning the screening of hypomania symptoms in outpatients with recurrent depression. Our study showed that 22% of outpatients were not diagnosed as bipolar disorder. Moreover, we found a significant association between undiagnosed bipolar disorder and the earlier age onset of depressive episode. In our study, outpatients who were screened positive for bipolar disorder did more than three depressive episodes, thought more about death and attempt to suicide. We found no significant association between bipolar disorder with other demographic characteristics and clinical features. These results could be explained by our relatively small size. Our first finding of the prevalence of the unrecognised bipolar disorder outpatients was 22%, was significantly lower than what was found in other studies where the rate was exceeding 40% [16]. The prospective studies carried out between 1970 and 1980 revealed that the prevalence of the unrecognised bipolar disorder varied from 0% to 40%, which consistent with our results [17, 18]. Also Rao et al [19] conducted a cohort study among depressed teenagers for 7 years; the results showed that 20% of participants had indeed the diagnosis of bipolar disorder. Another cohort of depressive outpatient without a history of bipolar disorder confirmed that 20% of them had actually bipolar disorder.

In our study, we found that wrongly diagnosed patient had an earlier age of onset of depressive episode. In fact, it's been demonstrate that young adults with early-onset major depressive disorder had a higher risk of progression to bipolar disorder [20–22]. One more study found that early onset of first depressive episode before age 25 was associated with cyclothymic temperament [23]. Goldberg et al [24] followed a group of young depressed patients during 15 years, they discovered that 41% had experienced an episode of mania or hypomania throughout the monitoring period. We found that 35% of undiagnosed outpatients did more than 3 depressive episodes, which is consistent with the results of other studies [25, 26]. These results affirm the interest of targeting patients with recurrent depression, these patients represent a privileged territory to have bipolar disorder, especially type II. Besides, bipolar disorder is characterized by a significant recurrence of major depressive episodes compared to unipolar depression [27]. The proportion of suicidal ideation and suicide attempts was higher among outpatients screened positive for bipolar disorder. Many studies have noted the significant association between suicidal potential and eventual bipolarity, compared to unipolar depression [15, 28, 29]. Early diagnosis and medical care of bipolar disorder patients can improve their quality of life. Mostly, the evolution of the illness becomes worse with time leading to damaged quality of life; indeed in our study the two-thirds of undiagnosed outpatients were unemployed and they had an unstable professional career [30, 31].

## Conclusion

The prevalence of the unrecognised bipolar disorder among outpatients with recurrent depression was similar to that found in other studies. The under-diagnosis bipolar disorder was associated with the earliest age onset of depressive episode and it was more prevalent in depressed patients with suicidal ideation and suicide attempts. These factors should be taken into account when we detect bipolar disorder, especially type II.

### What is known about this topic

- The high rate of the misdiagnosis bipolar disorder among outpatients with recurrent depression;
- The associated factors with the unrecognised bipolar disorder.

### What this study adds

- The first time in Morroco a study conducted to determine the prevalence of the unrecognised bipolar disorder;
- Our study showed some particular characteristics of a depressive episode among bipolar outpatients (having 3 depressive episodes were associated with BD);
- The outpatients with recurrent depression were the main focus of the study.

## Competing interests

The authors declare no competing interest.
